# Comparison of the Effects of Religious Cognitive Behavioral Therapy (RCBT), Cognitive Behavioral Therapy (CBT), and Sertraline on Depression and Anxiety in Patients after Coronary Artery Bypass Graft Surgery: Study Protocol for a Randomized Controlled Trial

**Published:** 2017-07

**Authors:** Seyed Hamzeh Hosseini, Alireza Rafiei, Ali Gaemian, Abdolhakim Tirgari, Aliasghar Zakavi, Jamshid Yazdani, Jafar Bolhari, Mahmood Golzari, Zahra Esmaeili Douki, Nazanin Vaezzadeh

**Affiliations:** 1 Psychiatry and Behavioral Sciences Research Center, Addiction Institute, Mazandaran University of Medical Sciences, Sari, Iran.; 2 Department of Psychosomatic, Imam Khomeini Hospital, Mazandaran University of Medical Sciences, Sari, Iran.; 3 Molecular and Cell Biology Research Center, Faculty of Medicine, Mazandaran University of Medical Sciences, Sari, Iran.; 4 Department of Cardiology, Faculty of Medicine, Mazandaran University of Medical Sciences, Sari, Iran.; 5 Department of Psychiatry, Member of Psychiatry and Behavioral Sciences Research Center, Sari, Iran.; 6 Department of Islamic Thought, Faculty of Medicine, Mazandaran University of Medical Sciences, Sari, Iran.; 7 Department of Biostatistics, Health Sciences Research Center, Faculty of Health Sciences, Mazandaran University of Medical Sciences, Sari, Iran.; 8 Mental Health Research Center (MHRC), Tehran Psychiatric Institute, Iran University of Medical Sciences (IUMS), Tehran, Iran.; 9 Faculty of Psychology and Educational Sciences, Allame Tabatabai University, Tehran, Iran.; 10 Department of Pediatric Nursing, Faculty of Nursing and Midwifery, Mazandaran University of Medical Sciences, Sari, Iran**.**

**Keywords:** *Religious Cognitive Behavioral Therapy*, *Cognitive Behavioral Therapy*, *Sertraline*, *Anxiety*, *Depression*, *Biomarker*

## Abstract

**Objective:** The present study aimed at comparing the effects of Religious Cognitive Behavioral Therapy (RCBT), Cognitive Behavioral Therapy (CBT), and sertraline on depression, anxiety, biomarker levels, and quality of life in patients after coronary artery bypass graft (CABG) surgery.

**Method:** This was a randomized controlled trial with parallel groups. A total of 160 patients after CABG surgery will be screened for anxiety and depression according to clinical interviews based on Diagnostic and Statistical Manual of Mental Disorders, Fourth Edition (DSM-IV) criteria and Hospital Anxiety Depression Scale (HADS) scores (≥ 8). To assess religious attitude, Golriz and Baraheni’s Religious Attitude questionnaire will be used. Participants will be randomly allocated to 4 groups of 40 including 3 intervention groups (RCBT, CBT, and sertraline) and 1 control group (usual care). RCBT and CBT programs will consist of 12 one-hour weekly sessions. The participants in the pharmacological intervention group will receive 25-200 mg/d of sertraline for 3 months. The Short Form-36 Health Survey (SF-36) will be administered to assess the patients’ quality of life. Blood samples will be taken and biomarker levels will be determined using the enzyme-linked immunosorbent assay (ELISA). The primary outcome will be reduction in anxiety and depression scores after the interventions. The secondary outcomes will be increase in quality of life scores and normalized biomarker levels after the interventions.

**Discussion: **If RCBT is found to be more effective than the other methods; it can be used to improve patients’ health status after CABG surgery.

Irct ID: IRCT201404122898N5

Coronary artery bypass grafting (CABG) is the first elective surgery for the treatment of acute coronary heart disease (1-2). While depression and anxiety are experienced by most patients after this type of surgery(3-5), studies have shown these 2 conditions to be associated with cardiac events (6), reduced quality of life(7), rehospitalization (8), and even death (9-10). 

A previous study indicated that an increase in depression symptoms reduced patients’ quality of life 6 months after 

heart surgery (11). Untreated depression and anxiety will negatively affect the prognosis of the disease(12) Furthermore, psychological problems are often accompanied by physiological changes. 

In this regard, higher levels of inflammatory cytokines (13-14) and lower levels of anti-inflammatory markers such as cortisol and dehydroepiandrosterone (DHEA) have been reported in patients with coronary artery disease and depression (15-16). However, contradictory findings have been discussed in various studies (17). 

Appropriate interventions to reduce depression and anxiety after CABG have been found to facilitate patients’ recovery and improve their quality of life (18-19). Selective serotonin reuptake inhibitors (SSRI), like sertraline, have been recommended as safe and well-tolerated treatment options for anxiety (20) and depression in coronary artery patients (21). In contrast, other studies argued that possible interactions between these drugs and other medicines used by older adults might cause some problems (22). Moreover, some patients prefer nonpharmacological interventions (23). Cognitive behavioral therapy (CBT) is an effective nonpharmacological intervention to treat depression in patients with physical problems (24) and cardiovascular diseases (25-26). Despite the efficacy of nonpharmacological interventions in reducing depression symptoms among patients with cardiovascular diseases (27), the existing evidence is insufficient (20, 23, 27). Furthermore, research has indicated the higher efficacy of nonpharmacological treatments with religious approaches in reducing depression and anxiety (28-31). However, the results showed different efficacies for conventional cognitive behavioral therapy (CCBT) and RCBT methods such that some studies have reported the same efficacies for CCBT and RCBT (32), and others found RCBT more effective than CCBT (33). Meanwhile, the results obtained showed that RCBT is slightly more effective than CCBT in more religious patients with depression and physical illness (34). In addition, a study showed that RCBT is not more effective than CCBT and that only in more religious people RCBT is more effective than CCBT in alleviating depression; however, this requires further studies (35). Considering the changes in the level of biomarkers of stress using RCBT and CCBT methods, one study revealed that neither RCBT nor CCBT was effective in obviating inflammatory cytokines, increasing anti-inflammatory cytokines, or reducing stress hormones in patients with chronic diseases and major depressive disorder. Moreover, in that study, CCBT had a greater effect only in reducing inflammatory cytokine IL-6 in less religious individuals, while RCBT had a greater effect in reducing IL-6 in more religious individuals (36). Further studies are required to determine whether religious interventions are able to change stress biomarkers in psychological disorders (37). Besides, these changes have been reported in a few studies that used nonpharmacological methods (38).

The primary aim of this study will be to compare the effectiveness of RCBT, CBT, and sertraline on anxiety and depression in patients post CABG surgery. The secondary aim of the study will be to compare the effectiveness of RCBT, CBT, and sertraline on patients’ quality of life and biomarker levels (IL-6, IL-10, DHEA, tumor TNF-α, cortisol) in patients post CABG surgery. This article describes the design and methods that will be used in the research

## Materials and Methods

A randomized controlled trial design with parallel groups will be adopted to compare the effects, RCBT, CBT, sertraline, and usual care on post-CABG anxiety and depression in patients admitted to Tuba Clinic, Sari, north of Iran. The participants who are referred to the clinic from all parts of Mazandaran province will be randomly allocated into 3 intervention groups (RCBT, CBT, and sertraline) and 1 control group (usual care). The study protocol was approved by the Behavioral Research Center of Mazandaran University of Medical Sciences (ID: 2013-12-04) and the Ethics Committee of the Research Deputy of Mazandaran University of Medical Sciences (ID: 92192). Moreover, all principles of the Declaration of Helsinki (2008) were considered during the study design.

***Inclusion and Exclusion Criteria:***

 The inclusion criteria are as follow: Age ≤ 65 years, having undergone first-time CABG surgery (4 weeks after surgery), having received follow-up treatment in Tuba Clinic, fluency in Farsi, junior high school degree, or higher level of education, willingness to participate and provide written consent, meeting the Diagnostic and Statistical Manual of Mental Disorders, Fourth Edition (DSM-IV) criteria for anxiety and depression (39), Score ≥ 8 for anxiety and depression on the Persian version of the Hospital Anxiety and Depression Scale (HADS)(40), and being religious (based on self-reports) (32). The exclusion criteria are as follow History of psychiatric disorders such as schizophrenia, bipolar disorder, and serious suicidal thoughts confirmed by clinical interviews performed by a psychiatrist based on the DSM-IV history of any physical conditions preventing the patients from participating in treatment sessions, history of attending RCBT, CBT, or any other types of psychotherapy programs, and using sertraline during the 2-week period prior to the study.

**Figure1:**
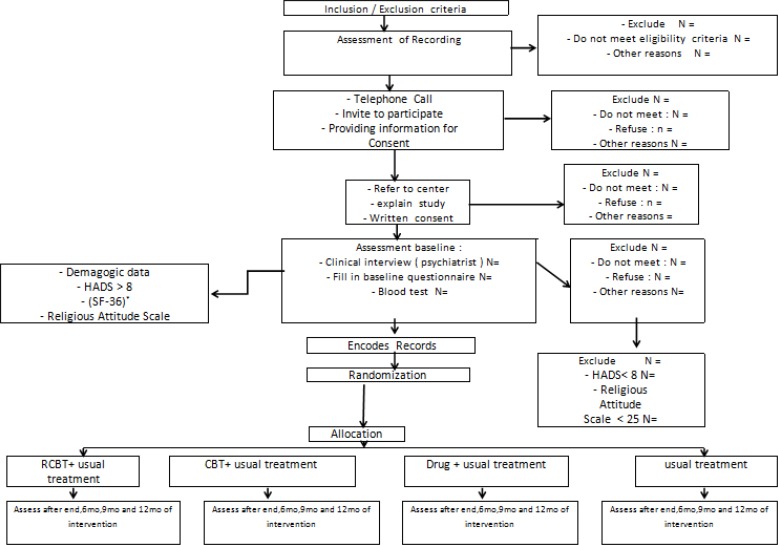
Flowchart of study design

1. ***Procedure*** **:** A researcher will first evaluate the records of patients who have undergone CABG in Tuba Clinic and select the eligible individuals based on the discussed inclusion and exclusion criteria. The selected patients will then be called and briefed about the project. Those who declare their willingness to participate during the phone call (oral consent) will be asked to refer to the clinic for baseline evaluations. During their visit, the participants will be asked if they regard themselves as a religious person (Yes/No). Individuals who provide positive answers will be asked to complete the Persian version of Golriz and Baraheni’s Religious Attitude Questionnaire (41). The patients will be included only if they score over 25 in the above-mentioned questionnaire. During the baseline evaluations, the participants will be reassessed with respect to the inclusion and exclusion criteria and explained about the project in detail. If the participants agree to participate, they will be asked to sign an informed consent form and will be randomized into one of the treatment conditions (conditions for discontinuation of this study and actions in that event treatment of subjects after this study is conducted). Then, to determine the presence of anxiety and depression symptoms, they will be asked to fill out the HADS. Individuals with HADS scores ≥ 8 will be referred to a psychiatrist to be interviewed based on the DSM-IV-TR. Participants with a number of mental disorders, e.g., schizophrenia, bipolar disorder, and serious suicidal thoughts will be excluded and referred to a psychiatrist to receive relevant treatment. The Short Form-36 Health Survey (SF-36) will then be administered to measure the participants’ quality of life (42). Blood samples will also be withdrawn, and enzyme-linked immunosorbent assay (ELISA) will be performed to determine IL-6, IL-10, TNF-ɑ, DHEA, and cortisol levels. After random allocation of the participants to the intervention and control groups, therapists trained in CBT and RCBT will perform the interventions. The participants will be evaluated at baseline (week 0), immediately after the interventions, and 6, 9, and 12 months after the interventions. Phone calls will also be made every 2 weeks to resolve any problems faced by the participants. Time intervals for measuring various outcome variables are summarized in Table 1. A flow chart of the study is presented in Figure 1.

Following the selection of eligible individuals using a random number table, a randomized block design, stratified by age (<57/>57) and gender (male/female) will be adopted to assign the participants to one of the 3 intervention groups or the control group (a total of 4 parallel groups; n = 40 each). The participants will be informed about their treatment groups after randomization. Participants will be recruited from Tuba Clinic, and multiple imputations will be used to handle attrition, ie, the sample size will be increased by up to 25% to remove attrition. Based on previous research (29), the sample size will be 160 patients (40 in each group) with α = 5% and test power = 80%.

At the first stage, the list of eligible participants for the study was extracted. Out of the 871 cases, 607 did not meet the inclusion criteria (77 cases provided wrong contact numbers, or missed calls, 7 cases died, 10 were unwilling to participate in the study, 175 could not participate due to the long distance, 292 did not have the age requirement, 6 had physical problems, 7 had heart valve surgery, and 33 were illiterate). Out of the remaining 264 patients, 104 patients who did not have the other criteria for entry were excluded from the study (47 cases due to their normal levels of anxiety and depression, 15 for their severe anxiety and depression, 32 due to their working conditions, 5 due to their reluctance to get the drug, and 5 cases due to their unwillingness to participate in group therapy sessions). The remaining 160 eligible patients were assigned into 4 groups: Religious cognitive behavioral therapy + usual care, cognitive behavioral therapy + usual care, drug group + usual care, and control group+ usual care.

A psychiatrist will conduct structured clinical interviews based on the DSM-IV. Clinical interview (DSM-IV-TR): This structured clinical interview has an acceptable reliability in clinical diagnosis (43).

 Patients will be excluded and referred to a psychiatrist for further treatment if they are diagnosed with bipolar disorder, schizophrenia, or serious suicidal thoughts. Patients with a history of psychiatric medications will also be excluded (39).

The Hospital Anxiety and Depression Scale (HADS): It assesses the possible presence of anxiety and depressive states and is considered to be unbiased by the presence of somatic illness and is found to be reliable and valid. Cronbach's alpha for HADS-A varies from .68 to .93 (mean .83) and for HADS-D from .67 to .90 (mean .82) (44). The reliability and validity of this scale was verified by Montazari et al. in Iran in 2003; its internal consistency with Cronbach alpha has been 0.78 for anxiety and 0.86 for depression (40).

The SF-36 is a 36-item questionnaire: It evaluates participants’ quality of life. The SF-36 has high internal consistency and good reliability and discriminate validity (Cronbach’s alpha above 0.70) (45). The validity and reliability of this scale in Iran has been confirmed. The questionnaire is arranged in 8 dimensions and has been reported to have a Cronbach’s alpha coefficient of 0.87 (42).

Golriz and Baraheni’s Religious Attitude Questionnaire comprises 25 items all scored on a 5-point Likert scale from 0 to 4 (the maximum total score is 100). The validity and reliability of this questionnaire were calculated to be 0.80 and 0.63, respectively (41). 

For the blood tests at the baseline and immediately after the interventions, 5-7 cc peripheral blood samples (in the form of clot) will be withdrawn by a laboratory assistant in Tuba Clinic (Sari). The samples will be used for IL-6, IL-10, DHEA, TNF-α, and cortisol measurements using ELISA. The serum will be immediately extracted, divided into little amounts, transferred to the laboratory of the Cellular and Molecular Biology Research Center, and maintained at -80 ºC until being tested. Similar transfer and processing procedures will be performed for all samples. The sandwich ELISA (an R&D kit, USA) will be applied to determine cytokine (TNF-α, IL-6, and IL-10) levels. Cortisol and DHEA levels will also be measured using ELISA.

***Ethics***

Before the initiation of the study, participants will be explained about the study objectives, the need for taking blood samples, and their right to withdraw at any stage. They will also be ensured about the safety of the interventions and confidentiality of data. The required permissions will be obtained from The Ethics Committee of Research Deputy of Mazandaran University of Medical Sciences and the relevant authorities at Tuba Clinic.

***Interventions*** ***:***

After the random allocation of the participants, the RCBT and CBT groups will attend 12 one-hour weekly sessions of therapy. The pharmacological intervention group will have to take 25-200 mg sertraline every evening for 3 months. The dose of the medicine will be determined based on the physician’s prescription. The control group will not receive any particular intervention. Usual care will be provided similarly to all groups.

***CBT*** ***:*** As an interactional approach, the CBT helps patients to identify the associations between thoughts, emotions, and behaviors. In this study, the patients will learn to adopt treatment strategies by making dynamic modifications to their cognition and behavior. They will also be trained to identify their automatic negative thoughts, evaluate their cognitions, and clarify their problematic behaviors. The subjects of each session will be provided based on the protocol along with the treatment techniques of Judith S. Beck (46). However, the original techniques will be tailored to the specific needs of CABG patients. A therapist and her assistant will hold 60-minute CBT sessions every week for 12 weeks. The appointment times will be set with the participants.

***RCBT*** ***:*** RCBT incorporates religious beliefs and motivations into the CBT process and uses them to encourage thought and behavior modifications (28). The principles of the technique have been adopted from Judith S. Beck’s cognitive-behavior model (46) and adjusted to fit religious beliefs and the sociocultural context of Iran. During their participation in an RCBT program, the patients are trained to modify their maladaptive beliefs, values, and behaviors and form a meaningful, hopeful, and optimistic view of the world (which is absent in depressed individuals) by using their own religious beliefs, doctrines, and behaviors. In fact, RCBT utilizes strong religious beliefs to encourage positive behaviors, which can ultimately eliminate depression (28). The modifications required for the needs of the current research were made based on the comments of a psychiatrist, a clinical psychologist, and religion experts. Similar to the CBT intervention, the RCBT will be performed by a therapist and her assistant over 12 one-hour weekly sessions scheduled with the patients.

***Pharmacological Intervention*** ***:***

The pharmacological intervention will involve the daily administration of 25-200 mg sertraline. Sertraline will be provided in the form of 50 and 100 mg pills by Abidi Pharmaceutical Co., Iran (licensed by Iran’s Ministry of Health). A physician will determine drug dose and ask the patients to take the medicine every evening for 3 months. Biweekly visits with the participants will be made during this period and the patients’ clinical management will be performed based on their responses to questions about the concomitant drugs and the experienced symptoms, side effects, and illnesses. Individuals who report any adverse effects will be excluded and referred to a psychiatrist for follow-up treatments. After the treatment period, the research assistant will call the patients every 2 weeks to follow on their health status and identify any existing problems. Patients with worsened symptoms will be excluded and referred to a psychiatrist for further treatment (based on ethical considerations).

***Usual Care*** ***:***Usual care will comprise physical care, medication, incision care (chest and leg), dietary recommendations, activities, and periodical visits after CABG surgery. All patients will be provided with an educational pamphlet containing the required information on routine care. Like the other groups, the control group will receive biweekly follow-up phone calls from the research assistant. Patients with worsened symptoms will be excluded and referred to a psychiatrist for further treatment (based on ethical considerations.

Classic Cognitive – Behavioral Therapy Sessions

*First Session:* Familiarity of members with each other, providing goals and rules of treatment sessions and patients expectations from the treatment

*Second Session:* Familiarity with patients’ problems, method of encountering them, experiences that patients have had in encountering problems, and learning diaphragm breathing and determining home assignments

*Third Session*: Reviewing previous session assignment and learning to recognize uncomfortable emotions, its motivating events and negative automatic thoughts, performing diaphragm breathing, and determining home assignments

Fourth Session: Reviewing the content and assignments of previous sessions and performing diaphragm breathing, progressive muscle relaxation, learning usage of techniques of challenging with negative thoughts, and determining home assignments

*Fifth, Sixth and Seventh Sessions:* Reviewing the content and assignments of previous sessions and performing diaphragm breathing, continuing learning progressive muscle relaxation, and continuing learning usage of techniques of challenging with negative thoughts, and determining home assignments

*Eighth Session:* Reviewing the content and assignments of previous session, learning cognitive distortion and their relation with automatic thoughts to patients, learning passive muscle relaxation, and determining home assignments

*Ninth Session:* Reviewing the content and assignments of previous sessions, continuing cognitive deviations and their relation with automatic thoughts of patients, passive muscle relaxation, and determining home assignments

*Tenth Session:* Reviewing the content and assignments of previous sessions and learning the patients' intermediate beliefs, passive muscle relaxation, and determining home assignments

*Eleventh Session:* Reviewing content and assignments of previous sessions, continuing learning intermediate beliefs, using techniques of challenging them, passive muscle relaxation, and determining assignments

Twelfth Session: Concluding subjects of previous sessions and its feedback

**Religious Cognitive – Behavioral Therapy Sessions**

***First Session:*** Familiarity of members with each other, providing goals and rules of treatment sessions, and patients expectations from treatment

*Second Session: *Familiarity with patients problems, method of encountering them, and experiences that patients have had in encountering problems (by their own religious beliefs) learning diaphragm breathing, and determining home assignments

Third Session: Reviewing previous session assignment, learning to recognize uncomfortable emotions, its motivating events and negative automatic thoughts, in addition to talking about faith in God and its effects on changing the automatic unpleasant negative thoughts, and performing diaphragm breathing and determining home assignments.

***Fourth Session:*** Reviewing the content and assignments of previous sessions, learning to use the techniques of challenging with negative thoughts, (talking about religious concepts of good and evil and enjoying it by challenging negative thoughts), performing diaphragm breathing, progressive muscle relaxation, and determining home assignments

Fifth , Sixth and Seventh Sessions: Reviewing the content and assignments of previous sessions, learning to use challenging techniques with negative thoughts (concepts of good and evil, discussing the concepts of patience), performing diaphragm breathing, progressive muscle relaxation, and determining home assignments

Eighth Session: Reviewing content and assignments of previous session, learning cognitive distortion and their relation with automatic thoughts to patients (discussions about miracles), progressive muscle relaxation, and determining home assignment. 

***Ninth Session:*** Review content and assignments of previous sessions and then continuing cognitive deviations and their relationship with automatic thoughts of patients (talking about relying on God power and man's relationship with God in worship, praise, and prayer), learning passive muscle relaxation, and determining home assignments

Tenth Session: Reviewing content and assignments of previous sessions, learning the patients' intermediate beliefs (talking about hope and trusting in God), passive muscle relaxation, and determining home assignments

Eleventh Session: Reviewing the content and assignments of previous sessions and continuing learning intermediate beliefs, using techniques of challenging them (talking about believing afterlife), passive muscle relaxation, and determining assignments

*Twelfth Session:* Concluding subjects of previous sessions and its feedback

***Data Analysis*** ***:***

In addition to descriptive statistics (mean, frequency, and percentage), inferential statistics, including t-tests, Chi-square tests, analysis of variance (ANOVA), Kaplan Meier, survival analysis, log-rank test, and Cox regression will be used to analyze the data. Analysis of covariance (ANCOVA) and regression analysis will also be applied to adjust the findings for the effects of age and sex. All analyses will be conducted in SPSS (Version 20).

## Discussion

Anxiety and depression have been reported as the most common psychological problems in patients with coronary artery disease after heart surgery, which may impair patients' physical and psychological functions. In psychiatric approach, many pharmacological and nonpharmacological therapies are used for treating psychological problems. This randomized controlled trial will attempt to compare the effects of RCBT, CBT, and sertraline on patients’ depression, anxiety, quality of life, and biomarker levels during a one-year period after CABG. If RCBT is found more effective than the other methods, it can be recommended to relieve anxiety and depression in CABG patients with drug intolerance or those interested in nonpharmacological approaches. Considering the religious background of most Iranians, RCBT can be administered to eliminate or reduce patients’ psychological problems and promote their health following CABG. This study will provide robust evidence on the effectiveness of treating anxiety and depression in CABG patients. It will also help to understand why this type of intervention might be helpful for these patients. 

**Table 1:** Measurement of outcome variables, at baseline (T1), after intervention (T2), 6 (T3), 9 (T4) and 12 (T5) month after intervention

**Variable **	**Measurement Tool **	**Time of assessment**				
		T1	T2	T3	T4	T5
Depression & anxiety	Hospital anxiety and Depression scale	*	*	*	*	*
Clinical interview	DSM-IV	*				
Religious atlitude	Golriz and Baraheni’s religious attitude (lranian’s version)	*				
Quality of life	Short Form-36	*	*	*	*	*
Blood Test (Biomarker: IL-6, IL-10,DHEA, TNF-α, cortisol)	ELISA	*	*			

The strengths of the study include: The effects of interventions will be assessed against those of usual care (the control group). Moreover, the long-term follow-up period will facilitate the long-term evaluation of the intervention effects. External validity, and thus outcome assessment of this clinical trial will be ensured by making direct comparisons between the 3 intervention groups and the control group. The findings of this study will have direct practical applications.

The follow-up of the treatment protocol by a number of therapists, administering reliable and valid tools, and determining the inter-rater reliability of the primary outcome will guarantee the internal validity of this clinical trial.

Finally, the participants’ efforts to support and interact with each other during the group therapy in CBT and RCBT programs will promote their self-confidence and lead to the development of senses of social competence and belonging.

## Limitations

Several limitations should be mentioned. In this study, blinding of the participants and therapists will be impossible. However, to minimize the effects of information bias, the statistician will not be informed about patient groups. A second drawback of the study will be the limited time allocated to each patient during RCBT and CBT sessions. Patients’ reluctance to discuss their problems in the group and their feelings of embarrassment will be the other clinical concerns during the study.

## Conclusion

Although recent evidence shows that pharmacological and nonpharmacological therapy are effective in treating anxiety and depression of patients with medical illness, few controlled clinical trials have evaluated the effects of religious CBT (RCBT) on the CABG patients. In summary, the result of the current study will reduce symptoms of anxiety and depression. Furthermore, it improves quality of life in this group of patients.
